# Reporting characteristics of non-primary publications of results of randomized trials: a cross-sectional review

**DOI:** 10.1186/1745-6215-14-240

**Published:** 2013-07-31

**Authors:** Sally Hopewell, Gary S Collins, Allison Hirst, Shona Kirtley, Abdelouahid Tajar, Stephen Gerry, Douglas G Altman

**Affiliations:** 1 Centre for Statistics in Medicine, University of Oxford, Botnar Recpsearch Building, Windmill Road, Oxford, UK; 2 INSERM U738 Paris, France; Centre d'Épidémiologie Clinique, AP-HP (Assistance Publique des Hôpitaux de Paris), Hôpital Hôtel Dieu, Paris, France

**Keywords:** Randomized controlled trial, Non-primary publication, Subgroup analyses, Secondary outcomes

## Abstract

**Background:**

For a randomized trial, the primary publication is usually the one which reports the results of the primary outcome and provides consolidated data from all study centers. Other aspects of a randomized trial’s findings (that is, non-primary results) are often reported in subsequent publications.

**Methods:**

We carried out a cross-sectional review of the characteristics and type of information reported in non-primary reports (*n* = 69) of randomized trials (indexed in PubMed core clinical journals in 2009) and whether they report pre-specified or exploratory analyses. We also compared consistency of information in non-primary publications with that reported in the primary publication.

**Results:**

The majority (*n* = 56; 81%) of non-primary publications were large, multicenter trials, published in specialty journals. Most reported subgroup analyses (*n* = 27; 39%), analyzing a specific subgroup of patients from the randomized trial, or reported on secondary outcomes (*n* = 29; 42%); 19% (*n* = 13) reported extended follow-up. Less than half reported details of trial registration (*n* = 30; 43%) or the trial protocol (*n* = 27; 39%) and in 41% (*n* = 28) it was unclear from reading the abstract that the report was not the primary publication for the trial. Non-primary publications often analyzed and reported multiple different outcomes (16% reported >20 outcomes) and in 10% (*n* = 7) it was unclear how many outcomes had actually been assessed; in 42% (*n* = 29) it was unclear whether the analyses reported were pre-specified or exploratory. Only 39% (*n* = 27) of non-primary publications described the primary outcome of the randomized trial, 6% (*n* = 4) reported its numerical results and 9% (*n* = 6) details of how participants were randomized.

**Conclusion:**

Non-primary publications often lack important information about the randomized trial and the type of analyses conducted and whether these were pre-specified or exploratory to enable readers to accurately identify and assess the validity and reliably of the study findings. We provide recommendations for what information authors should include in non-primary reports of randomized trials.

## Background

The Good Publication Practice for communicating company sponsored medical research (GPP2) guidelines [1] define a primary publication as the first full report of a study. For a randomized trial, the primary publication is the one which reports the results of the primary outcome (that is, the outcome used to determine the design and estimate the sample size of the randomized trial) and provides consolidated data from all study centers [2]. Additional findings from a randomized trial (that is, non-primary results) are frequently reported in subsequent publications. We refer to these types of publication as non-primary publications and might include pre-specified or exploratory subgroup analyses, perhaps analyzing only a specific subgroup of patients from the randomized trial, secondary outcomes, health economic analyses, or the patient outcomes after an extended period of follow-up [1,3]. Such publications are also sometimes referred to as secondary publications; however, this term can be misleading as it can be used to refer to summaries of existing studies or publications in other languages [4].

Readers of non-primary publications of randomized trials should be able to interpret the findings of these new analyses within the context of the previously published main results. However, there is limited evidence on how non-primary publications are reported in the literature or whether this might be improved [5]. In this study we describe the characteristics of a representative sample of published non-primary reports of randomized trials and assess the extent to which such publications report pre-specified or exploratory analyses. We also compared the trial information reported in non-primary publication with that reported in the full text of the corresponding primary publication.

## Methods

### Sample

We searched PubMed for all reports of randomized trials indexed from 1 July to 31 December 2009 with the publication type ‘Randomized Controlled Trial’ (search as of 4 January 2010). We limited our search to the National Library of Medicine’s (NLM) set of 121 Core Clinical Journals (formerly published as the Abridged Index Medicus), all of which are published in English.

### Eligibility criteria

We included all non-primary reports of randomized trials, which reported a comparison between patient groups. This comparison could be between intervention groups as randomized, as in the primary trial publication, or between groups not randomized, such as a comparison between patient subgroups, perhaps across interventions. We defined a non-primary publication as one, which reported trial results other than the primary trial publication (that is, the first publication with consolidated data from all centers, including the results for the primary outcome). We excluded non-primary reports of randomized trials that did not include a comparative analysis (for example, those exploring risk factors in a particular patient group) and those which reported early phase trials (for example, pilot and feasibility studies), trial protocols, or interim analyses.

### Screening process

One person (SH) screened the titles and abstracts of all retrieved reports to exclude any obvious reports of non-randomized studies. A copy of the full article was then obtained for all remaining records and two people assessed and confirmed whether or not they met the eligibility criteria. Any additional material about the trial included as an appendix on the journal website was also obtained if available.

### Data extraction

Data extraction was carried out by six reviewers working in pairs (in blocks of 25 articles allocated at random). Each reviewer independently extracted data from eligible reports; any differences between reviewers in a pair were resolved by discussion, with the involvement of an arbitrator if necessary. To ensure consistency between reviewers, we piloted the data extraction form using a sample of five papers from the sample under review. A data extraction manual was developed to provide guidance for each item on the data extraction form. Following piloting of the data extraction form the data extraction manual was modified slightly to ensure consistency in the data extraction process.

We extracted information on whether the comparison between treatment or patients groups was randomized, the journal type, source of funding, details of trial registration, reference to the trial protocol, whether the study was referred to as a non-primary publication in the abstract, the disease area and the type of intervention being investigated. We assessed the following study specific characteristics in relation to the non-primary publication: the number of study centers, the number of study groups, total sample size, and whether the non-primary publication analyzed all, or a subset of, randomized participants. We extracted data on the number of outcomes (where there was a comparison group) reported in the non-primary publication and, for the outcome which was the main focus of the non-primary publication, whether it was pre-specified or exploratory, the statistical methods used, how the results were reported and whether they were statistically significant. If more than one main outcome was reported we selected the one reported first in the methods section, but if not reported there we took the first outcome reported in the results section. We defined an outcome as a variable intended for comparison between groups; any outcomes assessed at multiple time points were classified as separate outcomes [6]. We also assessed the extent to which the non-primary publication reported information about the primary trial publication that reported the main results of the trial. Where non-primary publications provided a citation (or other unique identifier) to the primary publication of the trial, we compared the consistency of information reported in the two publications.

### Data analysis

All analyses were descriptive. The primary analysis focused on the general characteristics of the non-primary publication and the reporting of their study outcomes and results. We compared reporting between non-primary publications where the comparison between groups was or was not as randomized. We also compared the consistency of trial information reported in non-primary publications with that reported in the full text of the primary publication.

## Results

**Figure 1:**
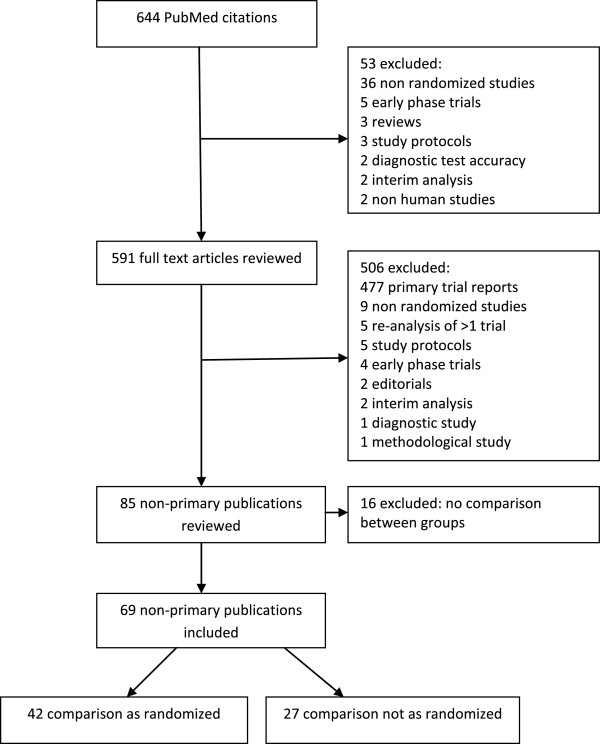
**Identification of non**-**primary publication from PubMed citations indexed from July to December 2009.**

The PubMed publication type search term ‘Randomized Controlled Trial’ identified 644 possible reports of randomized trials in the specified time window. After screening the titles and abstracts of all retrieved citations, we reviewed 591 full text articles (see Figure 1 for reasons for exclusion) resulting in 85 reports of non-primary publications; 16 were excluded as they did not include a comparison group. This resulted in 69 reports of non-primary publications; in 42 (61%) the comparison between groups was as randomized and in 27 (39%) the comparison was not as randomized. If a publication reported both types of comparison then we selected the one where the comparison between groups was as randomized.

### General characteristics of the non-primary publications

Table 1 provides information on the general characteristics of the non-primary publications. The majority (*n* = 56; 81%) of reports were published in specialty journals with by far the most common medical area being cardiology (*n* = 35; 51%). Forty-five percent (*n* = 31) of non-primary publication reports were non-industry funded, 25% (*n* = 17) were part industry funded and 14% (*n* = 14) were solely industry funded. Around half (*n* = 35; 51%) of the reports investigated drugs as the primary intervention of interest, whereas 32% (*n* = 22) assessed surgical or procedural interventions and 16% (*n* = 11) assessed counseling or lifestyle interventions. Details of trial registration (*n* = 30; 43%) or where the trial protocol (*n* = 27; 39%) could be accessed were each reported in less than half of non-primary publication reports. The majority (*n* = 56; 81%) of trials were described as multicenter, and most had two study groups (*n* = 55; 80%).

**Table 1:** **General characteristics of the non**-**primary publication**

	**Total *(n = *69)**	**Comparison as randomized *(n = *42)**	**Comparison not as randomized *(n = *27)**
Journal type			
Specialty	56 (81%)	30 (71%)	26 (96%)
General	13 (19%)	12 (29%)	1 (4%)
Funding source			
Solely industry	14 (20%)	9 (21%)	5 (18.5%)
Part industry	17 (25%)	12 (29%)	5 (18.5%)
Non-industry	31 (45%)	18 (43%)	13 (48%)
Unknown	7 (10%)	3 (7%)	4 (15%)
Trial registration			
Reported	30 (43%)	22 (52%)	8 (30%)
Not reported	39 (57%)	20 (48%)	19 (70%)
Trial protocol			
Reported	27 (39%)	17 (40%)	10 (37%)
Not reported	42 (61%)	25 (60%)	17 (63%)
Common disease specialties			
Cardiology	35 (51%)	16 (38%)	19 (70%)
Infectious diseases	3 (4%)	2 (5%)	1 (4%)
Rheumatology	3 (4%)	3 (7%)	0
Neurology	2 (3%)	1 (2%)	1 (4%)
Surgery	2 (3%)	1 (2%)	1 (4%)
Type of intervention			
Drug	35 (51%)	21 (50%)	14 (52%)
Surgery/procedure	22 (32%)	16 (38%)	6 (22%)
Counseling/lifestyle	11 (16%)	5 (12%)	6 (22%)
Equipment	1 (1%)	0	1 (4%)
Study centers			
Single	1 (2%)	0	1 (4%)
Multiple	56 (81%)	35 (83%)	21 (78%)
Unclear	12 (17%)	7 (17%)	5 (18%)
Main focus of non-primary publication			
Subgroup analyses	27 (39%)	11 (26%)	16 (60%)
Secondary outcomes	29 (42%)	19 (45%)	10 (37%)
Extended follow-up	13 (19%)	12 (27%)	1 (4%)
Time period assessed			
Reported	39 (57%)	23 (55%)	16 (59%)
Not reported	30 (43%)	19 (45%)	11 (41%)
Number of study groups in non-primary publication			
2	55 (80%)	33 (79%)	22 (82%)
3	10 (14%)	8 (19%)	2 (7%)
4	3 (4%)	1 (2%)	2 (7%)
≥5	1 (2%)	0	1 (4%)
Number of patients randomized in non-primary publication			
Median (IQR)	1,003 (347 to 2,699)	762 (256 to 2,659)	1,348 (660 to 2,928)
Range		34 to 20,479	
	34 to 21,906		36 to 21,906
Non-primary publication analyzed all randomized participants			
Yes	28 (40%)	20 (48%)	8 (30%)
No	33 (48%)	17 (40%)	16 (59%)
Unclear	8 (12%)	5 (12%)	3 (11%)
Flow diagram reported			
Yes	17 (25%)	9 (21%)	8 (30%)
No	52 (75%)	33 (79%)	19 (70%)
Identified as non-primary publication in the abstract^a^			
Yes	40 (59%)	23 (56%)	17 (63%)
No	28 (41%)	18 (44%)	10 (37%)

^a^Identified as non-primary in the abstract: publication did not have an abstract (*n* = 1).

Overall most non-primary publications reported on either subgroup analyses (*n* = 27; 39%), analyzing a specific subgroup of patients from the randomized trial, or reported on secondary outcomes (*n* = 29; 42%); 19% (*n* = 13) reported outcomes during extended follow-up periods (see Additional file 1 for more detail on the types of study identified). Non-primary publications where the comparison between groups was as randomized were more likely to report analyses of secondary outcomes (*n* = 19; 45%), whereas non-primary publications where the comparison was not as randomized were more likely to report on subgroup analyses or a specific subset of patients (*n* = 16; 60%).

### Reporting of non-primary publication study outcomes and results

Table 2 provides information of the type of outcomes and results reported in the non-primary publication. Just under half (*n* = 29; 42%) of non-primary publications reported between one and five different outcomes (where there was a comparison group), with 16% (*n* = 11) reporting >11 different outcomes and 16% (*n* = 11) reporting >20; poor reporting meant that the number of outcomes assessed was sometimes unclear (*n* = 7; 10%). It was also often unclear (*n* = 29; 42%) whether the analyses described in the non-primary publication were pre-specified (that is, planned and documented before examination of the data preferably in the study protocol) or exploratory (that is, the hypothesis being tested was not specified before examination of the data). However, the majority of non-primary publications did provide sufficient information on the statistical methods (*n* = 68; 98%) used to compare groups and reported a summary results for the main outcome of the non-primary publication (*n* = 66; 96%) with estimated effect sizes and precision (*n* = 52; 75%); in 64% (*n* = 44) the results for the main outcome were statistically significant.

**Table 2:** **Reporting of non**-**primary publication study outcomes and results**

	**Overall *(n = *69)**	**Comparison as randomized *(n = *42)**	**Comparison not as randomized *(n = *27)**
Number of outcomes reported in non-primary publication^a^			
1 to 5	29 (42%)	20 (48%)	9 (33%)
6 to 10	11 (16%)	5 (12%)	6 (22%)
11 to 20	11 (16%)	6 (14%)	5 (19%)
>20	11 (16%)	8 (19%)	3 (11%)
Unclear	7 (10%)	3 (7%)	4 (15%)
Outcomes reported in non-primary publication			
Pre-specified	22 (32%)	16 (38%)	6 (22%)
Exploratory	10 (15%)	7 (17%)	3 (11%)
Pre-specified and exploratory	5 (7%)	2 (5%)	3 (11%)
	32 (46%)	17 (40%)	15 (56%)
Unclear			
Analysis for main outcome of non-primary publication			
Pre-specified (in publication)	16 (23%)	12 (28.5%)	4 (15%)
	5 (7%)	5 (12%)	0
Pre-specified (in register)	19 (28%)	13 (31%)	6 (22%)
Exploratory	29 (42%)	12 (28.5%)	17 (63%)
Unclear			
Statistical methods used to compare groups for main outcome of non-primary publication			
Reported	68 (98%)	42 (100%)	26 (96%)
Not reported	1 (2%)	0	1 (4%)
Summary result for each group for main outcome of non-primary publication			
Reported	66 (96%)	40 (95%)	26 (96%)
Not reported	3 (4%)	2 (5%)	1 (4%)
Estimated effect size and precision of effect estimate for main outcome of non-primary publication			
Reported	52 (75%)	32 (76%)	20 (74%)
Not reported	17 (25%)	10 (24%)	7 (26%)
Results statistically significant for main outcome of non-primary publication			
Yes	44 (64%)	23 (55%)	21 (78%)
No	21 (30%)	15 (35%)	6 (22%)
Unclear	2 (3%)	2 (5%)	0
Not reported	2 (3%)	2 (5%)	0

^a^For which there was a comparison between groups.

### Reporting of information about the primary publication

It was unclear from reading the abstract in 41% (*n* = 28) of non-primary publications that it did not report the main results of the trial. Half (*n* = 33; 48%) of non-primary publications cited only the primary trial report, if additional publications were cited this usually related to the study protocol (*n* = 19; 27%); five only cited the trial protocol despite the main results of the trial having already been published (Table 3). Less than half (*n* = 27; 39%) of non-primary publications reported the main outcome of the trial (that is, that reported in the primary publication) with only 6% (*n* = 4) reporting its numerical results and only 9% (*n* = 6) reporting on the method of randomization.

**Table 3:** **Information about the primary publication reported in the non**-**primary publication**

	**Overall *(n = *69)**	**Comparison as randomized *(n = *42)**	**Comparison not as randomized *(n = *27)**
Number of citations relating to primary publication			
Cites protocol only^a^	6 (9%)	1 (2%)	5 (19%)
Cites primary publication only	33 (48%)	21 (50%)	12 (44%)
Cites protocol and primary publication	19 (27%)	12 (29%)	7 (26%)
Cites multiple publications	11 (16%)	8 (19%)	3 (11%)
Identified main outcome of primary publication			
Reported	27 (39%)	20 (48%)	7 (26%)
Not reported	42 (61%)	22 (52%)	20 (74%)
Numerical results reported for main outcome of primary publication			
Reported	4 (6%)	4 (10%)	0
Not reported	65 (94%)	38 (90%)	27 (100%)
Method of randomization			
Reported	6 (9%)	4 (10%)	2 (7%)
Not reported	63 (91%)	38 (90%)	25 (93%)

^a^Number of citations relating to primary publication: primary publication not published at time of non-primary publication (*n* = 1).

### Comparison of information between the non-primary and primary publication

Finally, we compared the information reported in the non-primary publication with that reported in the primary publication (Table 4). The majority (*n* = 56; 81%) of non-primary publications were published in specialty journals whereas most (*n* = 46; 68%) primary publications were published in general medical journals, with one-quarter (*n* = 16; 23%) having the same corresponding author in both publications. Non-primary publications were more likely to report being non-industry funded (Non-primary: 45% *versus* Primary: 36%) whereas primary publications were more likely to report being solely (20% *versus* 30%) or partially industry funded (25% *versus* 33%). The source of funding was the same in both the non-primary and primary publication 65% (*n* = 45) of the time. In seven publications, the source of funding was reported in the primary publication (solely industry funded *n* = 4; non-industry funded *n* = 3) but omitted from the non-primary publication. Less than half of non-primary and primary publications reported details of trial registration or where the trial protocol could be accessed.

**Table 4:** **Comparison of information between the non**-**primary and primary publication**

	**Non-primary publication *(n = *69)**	**Primary publication *(n = *69)**
Journal type		
Specialty	56 (81%)	22 (32%)
General	13 (19%)	47 (68%)
Funding source		
Solely industry	14 (20%)	21 (30%)
Part industry	17 (25%)	23 (33%)
Non industry	31 (45%)	25 (36%)
Unknown	7 (10%)	0
Trial registration		
Reported	30 (43%)	32 (46%)
Not reported	39 (57%)	37 (54%)
Trial protocol		
Reported	27 (39%)	26 (38%)
Not reported	42 (61%)	43 (62%)
Study centers		
Single	1 (2%)	3 (4%)
Multiple	56 (81%)	64 (93%)
Unclear	12 (17%)	2 (3%)
Time period assessed		
Reported	39 (57%)	64 (93%)
Not reported	30 (43%)	5 (7%)
Number of study groups		
2	55 (80%)	54 (78%)
3	10 (14%)	12 (17%)
4	3 (4%)	2 (3%)
≥5	1 (2%)	1 (2%)
Number of patients randomized		
Median (IQR)	1,003 (347 to 2,699)	1,452 (389 to 4,439)
Range	34 to 21,906	34 to 21,906

Most primary publications (*n* = 64; 93%) reported the time period in which the trial was conducted compared to around half of non-primary publications (*n* = 39; 57%). The median number of participants randomized in the primary publication was 1,452 (IQR 389 to 4,439, range 34 to 21,906). Only around one-third of publications (*n* = 26; 38%), reported the same number of participants in both the non-primary and primary publication. Just over half (*n* = 37; 53%) of primary publications reported a statistically significant result for main outcome of the trial. Interestingly, of the primary publications which reported a non-statistically significant result for the main outcome of the trial (*n* = 31; 45%), just over half (*n* = 17/31; 55%) reported the main outcome of the non-primary publication as being statistically significant.

## Discussion

### Summary of main findings

Our study provides an overview of the information currently reported in non-primary reports of randomized trials published in the scientific literature, the type of analyses they perform, and the extent to which they report information about the main outcome of the randomized trial and its results. The majority (*n* = 56; 81%) of non-primary publications were large, multicenter trials, published in specialty journals. Most reported on either subgroup analyses, analyzing a specific subgroup of patients from the randomized trial, or reported on secondary outcomes or analyses; a small number reported evaluating primary outcomes during extended follow-up periods. Less than half of non-primary publications reported details of trial registration, where the trial protocol could be accessed, or made it clear in the abstract that the report was not the primary publication for the trial. This could be misleading and make it difficult, or in some cases impossible, to identify multiple publications for the same trial [5,7].

Non-primary publications often reported results for multiple outcomes. It was frequently unclear, however, how many outcomes had actually been assessed or whether the analyses described in the non-primary publication were pre-specified or post-hoc exploratory analyses. Multiple testing, for example by performing multiple subgroup analysis, can be a problem because of the risk of false positive findings the more analyses that are performed [3,8]. This could be a particular problem for post-hoc analysis where it is often unclear how many analyses were undertaken and whether they were motivated by inspection of the data [9-11]. An investigator might also be tempted to ‘fish’ for, and selectively report, the results of statistically significant outcomes as opposed to non-significant outcomes [12]; thus one should be cautious in the interpretation of such results [13,14]. There is some indication of selective reporting in our study whereby primary publications, which reported a non-statistically significant result for the main outcome of the trial, were more likely to report the main outcome of the non-primary publication as being statistically significant.

Readers of non-primary publications of randomized trials should be able to interpret the findings of these new analyses in the context of the previously published main results. Authors should therefore provide sufficient details about the study methods, sample selection, the primary outcome, and its results to enable readers to assess the new findings [15]. However, in our sample less than half of non-primary publications reported the main outcome of the randomized trial, with very few reporting its numerical results.

### Comparison with other studies

We are not aware of other similar studies assessing the characteristics of and types of analyses reported in non-primary reports of randomized trials. However, several studies have examined the reporting of subgroup analyses published in primary reports of randomized trials (that is, reporting the primary outcome) and have identified similar shortcomings [9,11,16-18]. For example, Wang and colleagues [11] reviewed 97 primary reports of randomized trials published in the *New England Journal of Medicine* between 2005 and 2006; 59 (61%) reported subgroup analyses, with larger trials and multicenter trials being more likely to report subgroup analyses than smaller trials and single-center trials. Among the trials which reported subgroup analyses, only 21 (36%) mentioned these analyses in the methods section and in 40 (68%) it was unclear whether the subgroup analyses were pre-specified or exploratory. Assmann [9] reported similar findings in a review of 50 trials published in 1997 in four leading medical journals, as did Hernandez and colleagues [18] in a review of 63 cardiovascular trials published between 2002 and 2004.

### Study limitations

Our study has some limitations. First, we included only reports of non-primary publications identified in PubMed using the indexing term ‘Randomized Controlled Trial’. We will therefore have missed some non-primary reports that were not indexed using this term. The search was also limited to the National Library of Medicine’s set of Core Clinical Journals and so may not be representative of all journals. Second, we included only reports of non-primary publications which we identified as such from reading the abstract or full text of the article, we did not assess the trial protocol. Some non-primary publications may have been omitted where the distinction was not clear (that is, we identified them as primary reports) or where the authors changed the nature of the outcome from that specified in the protocol. For example Chan and colleagues [6] in a review of 102 reports of randomized trials, identified major discrepancies in the specification of outcomes when comparing the trial protocol with the published article. Given the limitations of our approach to this study, it is possible that we have underestimated the number of non-primary publications and the magnitude of the problem of poor reporting.

### Implications for practice

The CONSORT Statement, most recently updated in 2010, provides recommendations for reporting the findings of randomized trials [2]. While primarily aimed at reporting the primary results, it also gives some recommendations for when and how secondary outcomes and additional subgroup analyses should be reported within the context of the primary publication. We are not aware of any specific reporting guidelines for addressing non-primary reports of randomized trials (http://www.equator-network.org). In response to our findings, we identified some additional suggestions for what authors should describe when reporting these types of analyses in non-primary reports of randomized trials (see Table 5).

**Table 5:** **Recommendations for information to include in non**-**primary reports of randomized trials**^**a**^

**Item**	**Description**
Abstract	Objectives of this report, and whether analyses were pre-specified or exploratory. A statement that it is not the primary trial report
Objectives	Specific objectives or hypothesis of this report (for example, subgroup analyses, secondary outcomes, extended follow-up)
Methods	Set in context of main trial and its results, cite primary trial report, describe method of randomization, details of blinding (if done), completeness of follow-up, identify primary outcome, and summarize numerical results
Outcomes	Number and type of outcomes assessed in this report, and how and when measured. Whether outcomes were pre-specified or exploratory
Statistical methods	Statistical methods used to compare groups in this report
Participants	Number of intervention groups and whether this report includes all groups
	Number of participants randomized to each group and whether analyzed all randomized participants
Results	For each outcome a summary result and sample size for each group and the estimated effect size (for example, relative risk) and its precision
Other information	Registration number and name of trial registry
	Where the full trial protocol can be accessed, if available
	Sources of funding and other support (such as supply of drugs), role of funder

^a^Adapted from 2010 CONSORT Statement.

In particular, authors should make clear in the abstract that it is not the primary publication for the trial and whether the analyses being reported were pre-specified or exploratory analyses. In the full text of the article, authors should specify the objectives or hypotheses being tested for example whether reporting subgroup analyses, secondary outcomes, or extended follow-up, and the time point at which they are being assessed. It is also important to set the study objectives within the context of the main trial, giving details of the number of study groups, the interventions, key aspects of trial methodology including the method of randomization, and the primary outcome and its numerical results. Other important information includes the number of participants in each group and whether the non-primary publication analyzed all, or a subset, of randomized participants. The number of outcomes assessed in the non-primary publication, whether each analysis was pre-specified or exploratory, and the statistical methods used to compare groups should be reported. As in the main report, for each analysis authors should report a result for each group and the estimated effect size and precision. Details of trial registration and the trial protocol are also important to help readers have greater understanding of what was planned and what was done, and to assess the validity and reliability of the new findings within context of main randomized trial.

## Conclusion

Based on the findings from our study, it is clear that non-primary publications often lack important information about the randomized trial, the type of analyses conducted and whether these analyses were pre-specified or exploratory. Without such key information, it is difficult for readers to accurately identify such non-primary trial reports and to assess the validity and reliably of the study results. In response to our findings, we provide recommendations for what information authors should include in non-primary reports of randomized trials.

## Competing interests

The authors declare that they have no competing interests.

## Authors’ contributions

SH was involved in the design, implementation, and analysis of the study and in writing of the final manuscript. GSC, AH, SK, AT, SG, and DGA were involved in the design, implementation, and analysis of the study and in commenting on drafts of the manuscript. All authors read and approved the final manuscript.

## Supplementary Material

Additional file 1 **Examples of different types of analysis**^**a **^**reported in the non-primary publication.** Click here for file
