# Supplementary material for: Reporting characteristics of non-primary publications of results of randomized trials: a cross-sectional review
Source: Trials. 2013 Jul 31;14:240. doi: 10.1186/1745-6215-14-240 (PMC3733891; doi:10.1186/1745-6215-14-240)
Supplement: Additional file 1 — Examples of different types of analysisa reported in the non-primary publication. [file 1745-6215-14-240-S1.doc]

**Additional file 1.** **Examples of different types of analysis^a^ reported in the non-primary publication**

| **Subgroup analyses:** the non-primary publication assessed the treatment effect for a specific patient characteristics or specific subgroup of participants for either the primary or secondary outcome(s) of the randomized trial. Or the non-primary publication assessed a specific subset of participants for example analyzing data from a specific center(s) from within a multicenter trial. *Examples*: ‘*The EPHESUS study included 6*,*632 patients post*-*AMI with LVEF* ≤*40*% *and clinical HF or diabetes*, *receiving standard therapy*, *randomized to either eplerenone 25 mg*, *titrated to 50 mg daily*, *or placebo*, *with a mean follow*-*up of 16 months*. *Analyses of the length of stay and total number of days of HF hospitalizations per patient were conducted on a subgroup of 828 patients with subsequent HF hospitalizations*, *overall and across 5 distinct geographic regions*.’ [*212*] ‘*The purpose of the present study was to compare the bleeding and ischemic acute and late outcomes in patients aged* <*70 years to those in patients aged* ≥*70 years when treated using transradial coronary stenting and maximal antiplatelet therapy in the Early Discharge After Stenting of Coronary Arteries* (*EASY*) *trial*.’ [*222*] ‘*The COURAGE trial did not show a difference between PCI and OMT for the primary end point* (*death or myocardial infarction* [*MI*]) *during long*-*term follow*-*up*, *other important cardiovascular outcomes that could vary by treatment assignment in older versus younger patients were not previously reported*. *In the present study*, *we performed a post hoc subgroup analysis of baseline characteristics achieved treatment targets at 60 months of follow*-*up*, *and pre*-*specified cardiovascular outcomes during a 2*.*5 to 7 year* (*median 4*.*6 year*) *follow*-*up among patients age* <*65 versus* ≥*65 years at baseline*.’ [*448*] ‘*The Understanding Potential Long*-*Term Impacts on Function with Tiotropium* (*UPLIFT*) *study was a randomised*, *double*-*blind*, *placebo*-*controlled trial*. *5993 patients aged 40 years or more with COPD were randomly assigned to receive 4 years of treatment with either once daily tiotropium* (*18 μg*; *n*=*2987*) *or matching placebo* (*n*=*3006*). *In a prespecified subgroup analysis*, *we investigated the effects of tiotropium in patients with Global Initiative for Chronic Obstructive Lung Disease* (*GOLD*) *stage II disease*. *Primary endpoints were the yearly rates of decline in prebronchodilator forced expiratory volume in 1s* (*FEV1*) *and in postbronchodilator FEV1*, *beginning on day 30 until completion of double*-*blind treatment*.’ [*480*] **Secondary outcomes or analyses**: the non-primary publication assessed outcomes other than the primary outcome of the randomized trial. Or the non-primary publication performed secondary analyses of the primary outcome, for example per protocol analysis compared to intention to treat analysis. *Examples*: ‘*The main results of the COURAGE trial revealed no significant differences in the primary end point of all*-*cause mortality or nonfatal myocardial infarction* [*MI*]. *We sought to assess the impact of PCI when added to OMT on major prespecified tertiary cardiovascular outcomes* (*time to first event*) *namely cardiac death and composites of cardiac death*/*MI*, *cardiac death*/*MI*/*hospitalization for ACS*, *cardiac death*/*MI*/*stroke*, *MI*/*stroke*, *or cardiac death*/*MI*/*ACS*/*stroke*, *during study follow*-*up*.’ [*22*] ‘*The ExTRACT*–*TIMI 25 study*, *a large*, *randomized*, *multinational trial*, *demonstrated a reduction in death or nonfatal myocardial infarction when enoxaparin was used instead of UFH as adjunctive therapy for fibrinolysis in patients with STEMI*. *The present study evaluates short*- *and long*-*term cost effectiveness of using enoxaparin compared with UFH as adjunctive therapy for fibrinolysis in patients with STEMI*.’ [*450*] ‘*We performed secondary analysis of data from a multisite randomized*, *placebo*-*controlled clinical trial of antibiotics to prevent chorioamnionitis*-*associated mother*-*to*-*child transmission of HIV*-*1 and preterm birth in sub*-*Saharan Africa*. *Early neonatal morbidity and mortality were analyzed*. *In an intention*-*to*-*treat* (*ITT*) *analysis*, *infants born to women randomly assigned to antibiotics or placebo were compared* [*ref*]. *In addition*, *non*-*ITT analysis was performed because some women received non study antibiotics for various clinical indications*.’ [*200*] ‘*The study was a secondary analysis of the data collected during the Misoprostol Vaginal Insert Trial*, *a multisite*, *double*-*blind*, *randomized trial of women requiring cervical ripening before induction of labor*. *The primary outcome was to estimate the maternal and pregnancy characteristics that independently predict successful induction of labor*.’ [*190*] **Extended follow-up**: the non-primary publication assessed longer follow-up periods for either the primary or secondary outcome(s). *Examples*: ‘*The results of our multicenter randomized controlled trial comparing unilateral pallidotomy with bilateral subthalamic nucleus* (*STN*) *stimulation in advanced Parkinson disease* (*PD*) *demonstrated that STN stimulation is more effective than pallidotomy up to 1 year postoperatively*. *1*,*2 Here we present the 4*-*year follow*-*up of this trial*.’ [*186*] ‘*We conducted a randomized*, *controlled trial of a neonatal parenting intervention for very preterm infants and have previously reported that this did not improve maternal outcomes at three months corrected age*. *We have subsequently followed up this cohort to determine whether there are longer*-*term effects of intervention on infant outcomes*.’ [*157*] ‘*The REACT* (*Rescue Angioplasty Versus Conservative Treatment or Repeat Thrombolysis*) *trial randomized patients with failed thrombolysis to 1 of 3 groups*: *repeat thrombolysis*, *conservative therapy*, *or R*-*PCI*. *Primary end point outcomes to 6 months published in 2006 demonstrated a significant benefit for the R*-*PCI group*. *This study reports the 1*-*year major adverse cardiac and cerebrovascular events* (*MACCE*) *and late* (*up to 5 years*) *mortality for the REACT trial patients*.’ [*126*] |
| --- |

^a^*Analysis could be either pre*-*specified or exploratory*:

***Pre***-***specified analysis***: *the analysis or hypothesis being tested was planned and documented before any examination of the data*, *preferably in the study protocol*.

***Exploratory analysis***: *the hypothesis being tested was not specified before examination of the data*.
